# Enhancing Subjective Well-Being through Physical Activity for the Elderly in Korea: A Meta-Analysis Approach

**DOI:** 10.3390/ijerph17010262

**Published:** 2019-12-30

**Authors:** Doyeon Won, Jung-sup Bae, Hyun Byun, Kwang-bong Seo

**Affiliations:** 1 Department of Kinesiology, Texas A&M University-Corpus Christi, Corpus Christi, TX 78412, USA; 2 Department of Sport Science, Hanyang University-ERICA, Ansan 15588, Korea; 3 Department of Sport Industry Studies, Yonsei University, Seoul 03722, Korea; byunleo@gmail.com; 4 Department of Leisure, Sport and Taekwondo, Kyungmin University, Uijeongbu-si 11618, Korea; judoskb@hanmail.net

**Keywords:** physical activity, dose-response, subjective well-being, elderly, meta-analysis

## Abstract

The purpose of the current study was to meta-analytically investigate the psychological impacts of physical activity for the elderly population in Korea. The findings from 21 studies, using the comprehensive meta-analysis (CMA) program, indicated that participation in physical activity had a low, but meaningful, impact on the subjective well-being of elderly individuals. Of three exercise dose components, the duration of physical activity was the most influential, followed by the frequency and intensity of the physical activity. Of six subjective well-being measures, self-efficacy was most strongly associated with physical activity, followed by life satisfaction, leisure satisfaction, exercise satisfaction, successful aging, and happiness. Results of moderator analyses indicated that the influence of physical activity became more pronounced as the proportion of males increased. Meanwhile, of the three exercise dose components, only the relationship between the duration and subjective well-being was influenced as the percentage of participants without a spouse or partner increased. Overall, regular participation in physical activity is an effective way of promoting subjective well-being among older adults in Korea. Relevant guidelines regarding physical activity prescription and behavioral management strategies are discussed.

## 1. Introduction

According to the United Nations’ forecast, Korea is facing the biggest increase in the proportion of the elderly, with Korean society projected to become hyperaged by 2026 [1,2,3]. The rate of elderly population increase is faster than others, with the 65+ age group expected to make up more than 38% of the population in Korea by 2050 [2,3]. Trends in aging of the population are not uncommon, especially for Asian countries. To a lesser or similar extent, Asian countries as Singapore, Taiwan, China, and Thailand are also expected to become hyperaged societies in the near future [3]. Such demographic transitions might come with higher social and economic costs and issues in quality of life if not well-prepared. Thus, successful aging is a critical social and national issue for fast-aging countries such as Korea, given that aging is associated with decreased body functions and reduced income [4,5].

From the quality of life perspective, individual life quality is determined by several key factors, including physical well-being, relationships with other people, social activities, and recreation [6]. Among others, physical well-being is considered to be the most critical contributing factor, with positive spillover effects on the psychological, social, and material well-being of the elderly. Regular exercise or physical activities can benefit the elderly by enhancing physical self-efficacy and self-esteem and preventing a wide range of health problems and issues, including depression, cardiovascular disease, and osteoporosis [7,8]. Also, in the case of sport-based physical activities, the participant can achieve not only physical or health-related benefits, but also social and psychological benefits.

As stated, physical activities of the elderly should be considered as preventive and therapeutic actions as well as pursuits of happiness [9]. If physical health is not maintained, life quality and, thus, life satisfaction of the elderly person could be compromised [10]. Research suggests that those who regularly participate in physical activity at older ages are more likely to enjoy successful aging, in comparison to those who irregularly or intermittently participate [11,12]. Similarly, many empirical studies supported the positive impact of physical activity for the elderly. For example, Gopinath et al. reported that a high level of total physical activity, including the performance of moderate or vigorous activity and walking exercise, was positively associated with successful aging, such as the absence of depressed symptoms and systemic conditions [11]. Min found that elderly participation in sport-based leisure activities had a positive impact on their perceived self-esteem [13]. Similarly, Lee and Kim found that the duration of physical activity was positively associated with the life quality of older female adults [14].

Unfortunately, in Korea, the culture of physical activity among the elderly, especially in the form of (recreational) sport participation, is not well-established. Consequently, it is more common for the elderly to use medications and dietary supplements to maintain or enhance their health as opposed to utilizing sports medicine, i.e., physical activity prescription. The elderly Korean population is marginalized in terms of access to sport and leisure services due to socio-economic marginalization, urbanization, and population migration [15]. Consequently, the role and effect of physical activity on older adults in Korea might be substantially different to elderly people from other nations. In addition, many Korean older adults tend to believe information closer to what they know (e.g., studies conducted using Korean subjects). Thus, in order to provide more accurate guidelines for physical activity of the elderly population, it is important to understand to what extent physical activity is beneficial in attaining subjective well-being and which components of physical activity (i.e., the physical activity “dose”), namely, duration, intensity, and frequency, are strongly associated with subjective well-being.

However, some inconsistencies exist in regards to the effect of physical activity for the elderly Korean population. For example, Park and Ihm reported that all three components of physical activity, including the frequency, intensity, and duration, predicted the level of perceived life satisfaction [16]. However, Huh et al.’s study suggested that only the duration of physical activity had a positive influence on elderly life satisfaction, among others [17]. This inconsistency in results across studies might be, to a great extent, due to differences in sample characteristics, including gender ratio, physical activity types, socio-economic status, or subjective well-being measures. Also, some of the previous studies only measured part of the three physical activity components, while other studies analyzed gender-biased data. In this regard, a meta-analytic approach would resolve the contradicting or inconsistent results in regard to the influence of the three components of physical activity and the moderating roles of gender ratio and marital status, given that meta-analysis is a method that involves summarizing the results of related studies on a given topic [18]. As discussed, among other socio-demographic variables, the gender and marital or partnership status of older Korean adults could have a greater influence on their participation in physical activity. Korea has a strong Confucian culture, so-called “Korean Confucianism”; consequently, the legacy of Korean Confucianism has influenced the gender roles and Korean family life, especially in older populations. Therefore, it was deemed worthwhile to investigate the role of gender and marital status on the relationship between physical activity and subjective well-being of the elderly Korean population.

**Figure 1:**
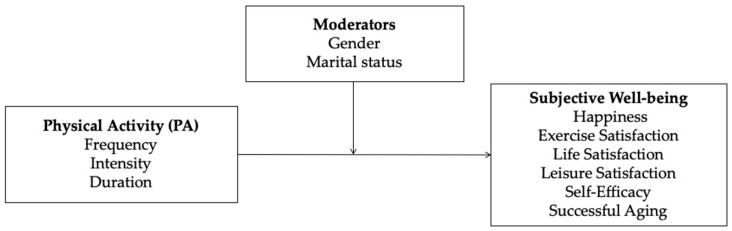
Research model.

Therefore, the primary purpose of the current study was to investigate whether the frequency, intensity, and duration of physical activity had effects on the Korean elderly’s subjective well-being. Specifically, this study aimed to investigate several research questions. First, this study aimed to evaluate the overall effect size of the elderly’s participation in physical activity on various dependent variables, including life satisfaction, successful aging, leisure and exercise satisfaction, self-efficacy, and happiness. Second, the influence of the three individual components of physical activities was investigated, namely the frequency, intensity, and duration of the physical activity, on a set of exercise benefits. Lastly, the moderating roles of gender and marital status on the relationship between physical activity and exercise benefits were explored. The research model of the study is presented in Figure 1. 

## 2. Materials and Methods

### 2.1. Data Sources and Search

**Figure 2:**
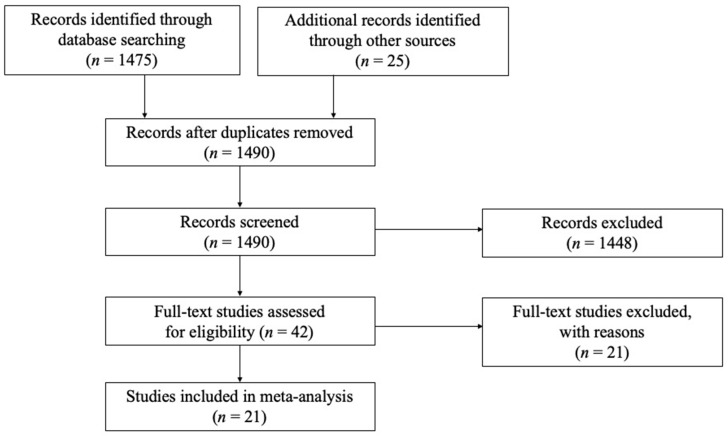
Flowchart outlining the study selection process.

Both published articles and unpublished theses and dissertations (covering 1995–2016) were included in the meta-analysis (see Figure 2). Duplicated unpublished studies were excluded when a certain thesis or dissertation was published in a journal. To find relevant studies, a search was conducted using three major national electronic databases, including PubMed, the National Assembly Library (NAL), Korea Education and Research Information Service (KERIS), and Korean-Studies Information Service System (KISS), using the keywords “physical activity for the elderly (in/for older adults)”, “exercise for older adults”, “sport participation among older adults”, and “leisure activity participation of older adults”. In addition, a reference list describing previous reviews of the related studies was inspected.

Two investigators independently reviewed all the titles and abstracts to identify potentially relevant articles and dissertations for further review. Disagreements between two investigators were resolved by a consensus. Studies were considered eligible if they measured either the frequency, intensity, and/or duration of study participants’ physical activities, included any kind of quality of life or subjective well-being measures, such as life satisfaction, successful aging, leisure satisfaction, exercise satisfaction, self-efficacy, or happiness, and included study participants aged 65 years and older.

As reported in Figure 2, an initial screening and subsequent screening yielded a total of 42 studies, including 22 journal articles and 20 dissertations. From the remaining studies, 21 studies were excluded if there was no relevant statistical information, such as zero-order correlation coefficients and data allowing computation of effect sizes [19,20]. The final sample for the current meta-analysis consisted of 21 studies (*N* = 7614), 12 journal articles and nine unpublished dissertations. Table 1 reports a list of the individual studies that were included in the meta-analysis.

### 2.2. Coding

To ensure the accuracy of coding, two of the authors coded all sample characteristics and relevant statistical data (e.g., estimates of the effects of physical activity and exercise) from each study. Differences, other than errors, were resolved by discussion with a third investigator when necessary. Coded items included the level of physical activity participation and outcome variables as well as the sample size, the gender proportion of the study, the percentage of the sample with a partner (married or cohabiting), publication type, and publication year.

The independent variable, physical activity dose, was coded in terms of the frequency, intensity, and duration. Also, the elderly’s subjective well-being (type) was coded into six categories, namely, exercise satisfaction, leisure satisfaction, life satisfaction, self-efficacy, successful aging, and happiness. The proportions of gender and marital status from each study were also coded. The type of marital status was coded as single (or divorced) or married (or with a domestic partner). In addition, correlations between physical activity and subjective well-being (i.e., physical activity-related outcomes) were coded for each study.

### 2.3. Statistical Procedures

A random-effects meta-analysis was conducted using Comprehensive Meta-Analysis (CMA) software [37]. A correlation between variables was considered significant if the 95% confidence interval (CI) excluded zero. The stability of the effects was tested using the Fail-Safe sample size (*N*) [38]. A result of the meta-analysis was considered robust to publication bias if the effect size is greater than “5K + 10”, where *k* is the number of studies [38]. Hunter and Schmidt’s Q test was used to test the significance of the estimated variance of the population correlation [39]. Moderator effects were tested using *z*-tests to examine the differences in the weighted correlations [39].

## 3. Results

### 3.1. Aggregated Effect of Physcial Activity on Subjective Well-Being

**Figure 3:**
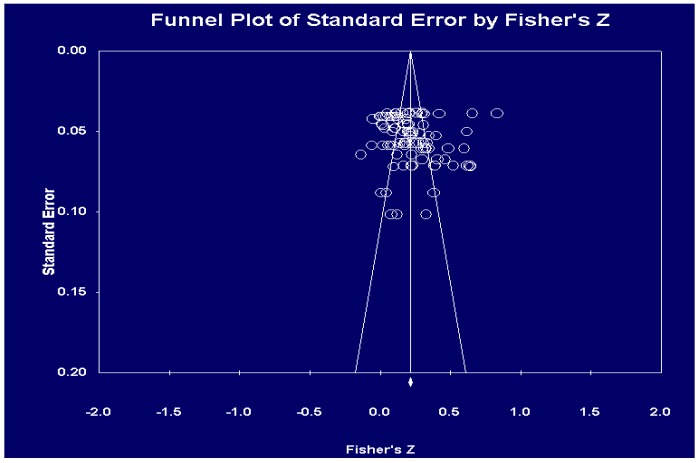
Funnel plot.

The mean correlation between physical activity and individual-level psychological outcomes (aggregated over outcomes) was *r* = 0.21, 95% CI = [0.17, 0.26], *p* < 0.001, which showed a small but significant positive effect of physical activity across the subjective well-being range among the elderly Korean population (see Table 2). Effect sizes ranged from 0.096 to 0.338 throughout the 21 studies. Heterogeneity was considerable, with a high *I*^2^ value of 91.77%, indicating the potential presence of moderators for the physical activity effect on the elderly’s subjective well-being [40]. There was no conclusive evidence of sample bias (Egger’s Test of the Intercept *B*_0_ = −0.028, *p* > 0.05), and the funnel plot of standard error and log rate ratio was fairly symmetrical (see Figure 3). The Fail-safe *N* was 4070, suggesting that there would need to be an additional 4,070 studies added to the analysis in order for the cumulative effect to become significantly nonsignificant.

As shown in Table 2, the mean correlations between physical activity and six psychological outcome measures were also calculated: life satisfaction (*k* = 9), leisure satisfaction (*k* = 5), self-efficacy (*k* = 5), happiness (*k* = 4), successful aging (*k* = 3), and exercise satisfaction (*k* = 1). The strongest correlation was found with self-efficacy, *r* = 0.34, 95% CI = [0.31, 0.37], followed by life satisfaction, *r* = 0.21, 95% CI = [0.19, 0.23], leisure satisfaction, *r* = 0.18, 95% CI = [0.13, 0.23], exercise satisfaction, *r* = 0.13, 95% CI = [0.08, 0.18], successful aging, *r* = 0.13, 95% CI = [0.09, 0.16], and happiness, *r* = 0.10, 95% CI = [0.06, 0.13]. Only the effect of physical activity for self-efficacy was found to be moderate [41].

### 3.2. The Duration, Frequency, and Intensity of Physcial Activity on Subjective Well-Being

The effects of physical activity’s three components on subjective well-being were examined (Table 3). The correlation was stronger with the duration of physical activity (*r* = 0.27), followed by the frequency (*r* = 0.20) and intensity (*r* = 0.17). The 95% confidence interval for all three components did not include zero, indicating that all three correlations were statistically significant. 

The effects of the three PA components on each subjective well-being measure were also examined. As expected, the results were almost identical to the aggregated effect of physical activity. The effects of the duration, frequency, and intensity were stronger on self-efficacy (*r* = 0.37, *r* = 0.31, and *r* = 33, respectively) than other subjective well-being measures. Only the intensity did not exhibit a significant effect on leisure satisfaction and happiness.

### 3.3. Moderator Analyses

**Figure 4:**
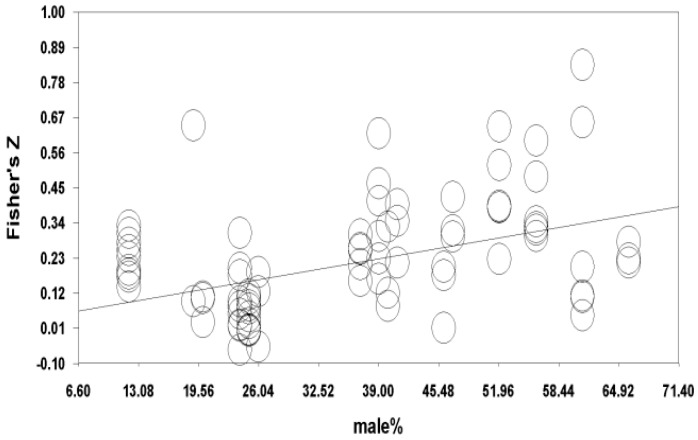
Fisher’s Z by the ratio of male subjects.

**Figure 5:**
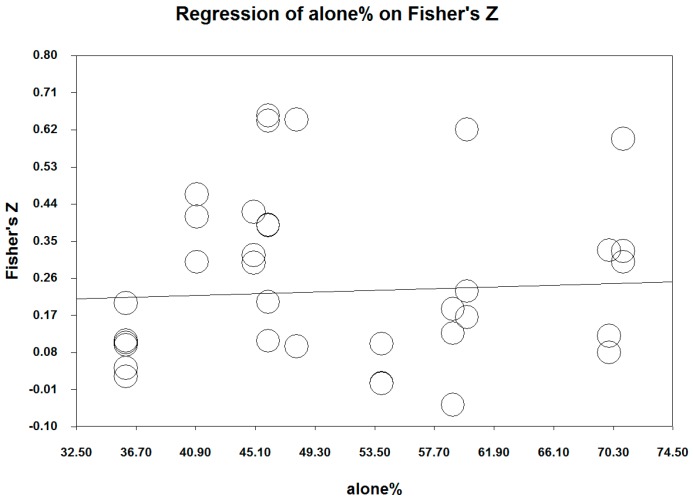
Fisher’s Z by the “absence of spouse” ratio.

Moderator analyses using meta-regression were performed based on the ratio of male subjects (Table 4 and Figure 4) and the ratio of the absence of a spouse/partner (Table 5 and Figure 5) per each study, given the suggested heterogeneity in this study. For gender differences, the ratio of male subjects (percentages of male participants) per study was entered as a predictor of the effect sizes, in order to examine whether the relationship between physical activity and the aggregated subjective well-being was influenced by the proportion of gender in each study. The results indicated that the aggregated influence of physical activity was more pronounced as the proportion of males increased (*z* = 10.08, *p* < 0.001). Of the three components of physical activity, the influence of the duration (*z* = 11.69, *p* < 0.001) and intensity (*z* = 7.13, *p* < 0.001) on the aggregated subjective well-being increased as the proportion of male subjects increased. However, the gender proportion did not influence the relationship between the frequency and the aggregated subjective well-being.

Secondly, the percentage of those living without a spouse or partner (i.e., the absence of a spouse) was entered as a predictor of the effect sizes. Counter to what might be expected, the influence of the duration increased as the percentage of participants without a spouse or partner increased (*z* = 2.23, *p* = 0.025). Regarding the other components, the absence of a spouse did not moderate the relationship between the frequency/intensity and the aggregated subjective well-being.

## 4. Discussion

The current meta-analysis showed small to moderate, but significant, effects of physical activity on the subjective well-being of the Korean elderly population. In particular, physical activity had a stronger influence on the self-efficacy of the elderly, among others. The results suggested a positive association between physical activity and subjective well-being, which was consistent with the previous studies [42]. Many elderly people in Korea tend to rely on dietary and health medicines and supplements to promote and maintain their health, preferring non-physical activities to promote socio-psychological well-being, as opposed to physical leisure and recreational activities. However, the results of the current study demonstrated the efficacy and utility of physical activity in the promotion of elderly Koreans’ subjective well-being. Overgaard et al. argued that physically active individuals who consumed energy- and nutrient-balanced diets did not gain additional benefits on health from the consumption of dietary supplements [43]. Therefore, the concept of physical activity prescription should be embraced by health and human services agencies in Korea.

Of the various subjective well-being indicators, the self-efficacy of elderly people could be enhanced by physical activity to a greater extent, followed by life satisfaction, leisure satisfaction, (perceived) successful aging, and happiness. Thus, the results indicated that all subjective well-being measures were positively affected by participation in physical activities. Consequently, health policymakers and practitioners should promote physical activity among the elderly, for example, by providing proper exercise prescription. According to Mazzeo and Tanaka, there are five exercise prescription elements, i.e., warm-up, exercise intensity, exercise duration, frequency, and exercise type/mode [44]. In most cases, elderly Koreans do not receive proper exercise prescription due to the lack of guidelines for medical or sport medicine professionals. The results of the current study suggested that, of the three exercise dose indicators, the duration of physical activity was the most important aspect, followed by the frequency and intensity of physical activity, in the promotion of the subjective well-being of the elderly person. Per exercise prescription guidelines [44], future studies should also examine the efficacy and influence of physical activity type/mode on the elderly’s subjective well-being and other health benefits.

According to the American College of Sports Medicine (ACSM)’s guidelines concerning physical activity programs for older adult populations [45], there are seven factors that influence behavioral management strategies in physical activity interventions, including social support, self-efficacy, active choices, health contracts, perceived safety, regular performance feedback, and positive reinforcement. Given the results of the current study, a behavioral management strategy to expedite initial participation and sustain continuous participation in physical activity should be developed to address the “duration” aspect of the physical activity.

Of the six subjective well-being measures investigated in this study, especially, self-efficacy is a factor that increases self-esteem and reduces anxiety levels, and it can be enhanced by participation in physical activity. On the other hand, self-efficacy is one of the factors that positively influences effective behavioral management strategies in physical activity interventions [45]. Thus, it can be argued that elderly participation in physical activity triggers a virtuous cycle in the development of physical activity-based health promotion.

The results of our moderator analyses indicated that elderly males and those without a spouse or partner materialized the psychological benefits of physical activity to a greater extent. These results were somewhat related to the male-gatekeeping, gendered culture in sports and physical activity in Korea, where elderly women experience a greater level of participation constraints and difficulty in accessing physical activity. Therefore, policymakers should develop strategies to support facilitators and reduce participation barriers to promote the physical activity-based healthy lifestyle of older adults [46].

There are some noteworthy limitations in this study. As previously stated, the current study purposefully examined the previous studies conducted in Korean populations. Therefore, the results of the current study may not be generalized to other contexts, or there might be some different dose–response relationships between physical activity and subjective well-being measures. In addition, the current study only included studies utilizing a cross-sectional research design, as opposed to experimental studies. Thus, future studies should consider conducting a meta-study on experimental studies concerning the dose–response relationship. Furthermore, the current study conducted moderator analyses using the proportion of gender and marital status per study. Consequently, future studies should consider other socio-economic variables as moderators in the relationship between physical activity and subjective well-being.

## 5. Conclusions

In Korea, many older adults believe that dietary supplementation is a better way to maintain their health; elderly people have limited confidence in the health benefits of physical activity. However, the results of the current meta-study clearly indicate that regular physical activity brings psychological health benefits to older adults. In particular, physical activity in elderly people brings a greater level of subjective well-being in terms of self-efficacy, which is an important element of sustainable participation in physical activity. Of the three physical activity doses, the duration of physical activity is the most critical element when designing a physical activity prescription for older adults. In the Korean context, male older adults and elderly people without a spouse or partner experience higher levels of physical activity-related benefits. Consequently, health and human services agencies should develop policies and invest resources to maximize the benefits of physical activity for elderly women and couples.

## Figures and Tables

**Table 1:** Studies included in the meta-analysis.

ID	Author	Year	Type	Sample Size	Dependent Variable(s)
1	An [21]	2002	Dissertation	200	Leisure satisfaction
2	Choi [22]	2016	Dissertation	310	Happiness, self-efficacy, quality of life
3	Hong [23]	1996	Dissertation	100	Life satisfaction
4	Huh, Jung, and Ha [17]	2007	Journal	244	Life satisfaction
5	Hwang [24]	2004	Dissertation	223	Life satisfaction
6	Hwang [25]	2015	Dissertation	197	Successful aging
7	Jeon [26]	2010	Journal	276	Self-efficacy, happiness
8	Kang and Cha [27]	2010	Journal	612	Leisure satisfaction, life satisfaction, happiness
9	Kim [28]	1995	Journal	400	Leisure satisfaction, life satisfaction
10	Kim [29]	2010	Dissertation	475	Happiness
11	Kim [30]	2016	Dissertation	385	Successful aging
12	Kim and Yoon [12]	2007	Journal	490	Exercise satisfaction
13	Lee [31]	2004	Journal	683	Leisure satisfaction, life satisfaction
14	Lee [32]	2015	Dissertation	566	Successful aging
15	Lee and Kim [14]	2009	Journal	132	Life satisfaction
16	Lee, Kim, and Kang [33]	2012	Journal	294	Leisure satisfaction, self-efficacy
17	Min [13]	2011	Journal	664	Self-efficacy
18	Park and Ihm [16]	2008	Journal	670	Self-esteem, life satisfaction
19	Park and Ku [34]	2005	Journal	200	Leisure satisfaction, life satisfaction
20	Shin [35]	2013	Dissertation	439	Happiness
21	Yi, Ahn, and Sim [36]	2011	Journal	364	Self-efficacy

**Table 2:** Effect sizes of physical activity on all subjective well-being outcomes.

Dependent Variables	Model	ESr (r)	−95% CI	+95% CI	Q	I^2^	SE	Fail-Safe *N*
Aggregated	Fixed (Random)	0.200 (0.212)	0.188 (0.167)	0.213 (0.255)	704.399	91.766	0.006	4070
Self-efficacy		0.338	0.310	0.365	30.962	74.162	0.005	
Life satisfaction		0.210	0.191	0.229	329.133	92.100	0.011	
Leisure satisfaction		0.184	0.134	0.233	50.917	90.180	0.027	
Exercise satisfaction		0.129	0.079	0.179	10.378	80.729	0.011	
Successful aging		0.125	0.091	0.159	129.250	94.584	0.026	
Happiness		0.096	0.059	0.133	8.986	44.357	0.003	

*Note*: ESr (*r*) = Effect size correlation coefficient; CI = Confidence interval; Q = Cochran’s Q; *I*^2^ = inconsistency index (% of variation across studies that is due to heterogeneity rather than chance).

**Table 3:** Effect sizes of three sub-components (exercise doses).

Physical Activity Participation	Dependent Variables	ESr	−95% CI	+95% CI	Q	I^2^	SE
Duration	Psychological outcomes (aggregated)	0.265 (0.269)	0.141 (0.089)	0.286 (0.339)	254.227	92.526	0.013
Frequency		0.171 (0.195)	0.244 (0.195)	0.192 (0.269)	245.859	92.272	0.012
Intensity		0.163 (0.168)	0.149 (0.119)	0.186 (0.244)	151.415	88.112	0.008
Duration	Life satisfaction	0.285	0.253	0.317	150.335	94.679	0.032
	Successful aging	0.200	0.143	0.255	4.746	57.859	0.007
	Leisure satisfaction	0.277	0.193	0.357	37.526	97.335	0.226
	Self-efficacy	0.371	0.323	0.417	17.557	88.609	0.022
	Happiness	0.153	0.089	0.215	2.055	51.349	0.006
Frequency	Life satisfaction	0.185	0.151	0.218	91.001	91.209	0.019
	Successful aging	0.100	0.042	0.157	96.152	97.920	0.144
	Leisure satisfaction	0.195	0.109	0.279	0.319	0.000	0.006
	Self-efficacy	0.310	0.260	0.359	0.369	0.000	0.003
	Happiness	0.072	0.007	0.136	0.693	0.000	0.003
Intensity	Life satisfaction	0.157	0.123	0.190	54.968	85.446	0.011
	Successful aging	0.064	0.001	0.128	17.330	94.230	0.054
	Leisure satisfaction	0.077	−0.011	0.165	2.556	60.869	0.015
	Self-efficacy	0.331	0.282	0.379	9.920	79.839	0.013
	Happiness	0.064	−0.001	0.128	1.733	42.280	0.005

**Table 4:** Results of meta regression analysis with the ratio of male subjects.

	Classification	Estimate	SE	−95% CI	+95% CI	*z*-Value	*p*-Value
Duration	slope	0.00986	0.00084	0.00821	0.01152	11.69237	0.00000
	intercept	−0.11136	0.03517	−0.18030	−0.04242	−3.16599	0.00155
Frequency	slope	−0.00103	0.00084	−0.00268	0.00062	−1.22034	0.22233
	intercept	0.21568	0.03517	0.14674	0.28462	6.13184	0.00000
Intensity	slope	0.00620	0.00087	0.00449	0.00790	7.13147	0.00000
	intercept	−0.06350	0.03665	−0.13534	0.00833	−1.73259	0.08317
Total	slope	0.00496	0.00049	0.00399	0.00592	10.0824	0.00000
	intercept	0.01618	0.02058	−0.02416	0.05651	0.78602	0.43186

**Table 5:** Results of the regression meta-analysis alongside the “absence of spouse” ratio.

	Classification	Estimate	SE	−95% CI	+95% CI	*z*-Value	*p*-Value
Duration	slope	0.00331	0.00148	0.00041	0.00621	2.23553	0.02538
	intercept	0.12914	0.0799	−0.01587	0.27416	1.74546	0.08090
Frequency	slope	0.00016	0.00148	−0.00274	0.00306	0.11029	0.91218
	intercept	0.19118	0.07399	0.04617	0.33620	2.58398	0.00977
Intensity	slope	0.00046	0.00148	−0.00337	0.00244	−0.31391	0.75359
	intercept	0.21115	0.07413	0.06586	0.35645	2.84836	0.00439
Total	slope	0.00100	0.00085	−0.00068	0.00267	1.16702	0.24320
	intercept	0.17793	0.04274	0.09416	0.26171	4.16274	0.00003
